# Annotations

**Published:** 1891-04-04

**Authors:** 


### Annotations.

At the beginning of a holiday nobody thinks of diet; at the end of it some people cannot think of anything else. One of the most important elements of a holiday is pleasure; but the man of work must think of the profit of a few days' change as well as of the pleasure. If he does not he often finds himself wishing at the end of his brief excursion that he had stayed at home.

**Holiday Diet.**

What is the proper diet for a holiday? Should the food be rich, varied, and abundant, or should it be limited both in quantity and variety? The average Englishman loves good living, and in ordinary circumstances it suits him. As a general rule, if the table be fairly generous at home, it may be a little more generous during a holiday. But many people are not content with a little increase of generosity at the table. They pass *per saltum* from one egg at breakfast, and one piece of bacon, to two pieces of bacon and three eggs. Instead of their usual light luncheon at midday they take a dinner of three or four courses, and perhaps half a bottle of wine. In the evening the dinner proper is turned into a gourmand's feast; two or three kinds of wine are partaken of freely; and the "feed" is followed by a glass or two of whiskey and water in the smoking room, and an extra cigar. For one or two days all goes well. The fresh air, the cheerfulness, the absence of work and worry help the digestive organs to rise to the increased demands made upon them, and to do their work heroically. But even the stomach cannot always be in a heroic mood; and when it finds that the only result of its efforts is that its owner makes still larger demands upon its energies, it first sulks and then strikes. Here is where a little medical knowledge would be useful. At the end of two or three days, when the first signs of discomfort are being felt, if an immediate change of diet be adopted, and a good strong liver pill taken, all bad consequences may be averted. But if such knowledge be wanting, the liberal diet is generally persisted in, and perhaps the whiskey or the brandy is increased. At last comes the catastrophe in the shape of a sharp bilious attack, with headache, fever, and general misery. What then is the really judicial and philosophic rule of diet for a short holiday? *Festina lente* is the golden rule. Change the diet slowly and gradually. Do not rush from the moderate table at home to a series of dinner-luncheons at midday and city company dinners in the evening. Move forward a step at a time. Then the digestion will improve as well as the appetite during the whole holiday, and when the return home is made, the increased appetite and power of digestion may give increased energy and capacity for work for many weeks to come.

**Holiday Quiet.**

The poor washerwoman, who had reached the age of seventy without being able to retire on an adequate pension, had a very definite idea of heaven. Her settled conviction was that in that blissful sphere she was "going to do nothing for ever and ever." Many people seem to spend all their lives in doing nothing, and yet, if you ask them, they will tell you that "doing nothing" is their most deadly aversion. But to the really hard-worked man, whose strenuous hours are filled with exacting toil, and to whom the loss of even a single hour in the week means a serious dislocation of business—to him, the most delicious of all the feelings that brighten his brief holiday is that of having nothing at all to do. Nobody wants to see him on urgent business; he has neither letters nor leaders to write. The collar is off, and the saddle, and even the halter. If he wants to walk he can walk; if he chooses to sit he can sit; if he prefers to lie down he can lie. He is not the sort of madman who tears about on a fresh excursion to a country house, a picture gallery, a waterfall, or a village sack-race every day. Though, perhaps, if he is very tired he may indulge a sneaking longing for the sack-race. It is change he wants; but change which involves no mental exertion: change which is rest. Modern life is exacting, and when not lived with intelligent discretion, killing. We can, most of us, work hard, and some have great mental powers; but it is the physiological financier who wins the day in the long run. "Any fool can make money," says the Yorkshire proverb; "but it takes a wise man to spend it." And so, a very ordinary man may do brain work, and good brain work; but it takes a man of unusual sense so to use his brains, in these competitive times, as to continually improve them, and to find himself richer in brain power at seventy that he was at twenty-seven. But this is what ought to be. A man should dread brain poverty for his old age exactly as he dreads financial poverty. For hard workers, rest is the chief thing: rest and quiet. Not merely change of occupation, but absolute laziness and "lying fallow." The gospel of holidays is the salvation of hard workers. So long as they are sufficiently numerous and sufficiently long, a man may work almost as hard as he pleases; it is impossible for him to kill himself. The most profitable investment to which a hard-worked man can put his money is a good holiday. But then it must be a real holiday; not a holiday that knocks up the legs and the stomach at the same time. The art of judicious holiday-making is still in its infancy in England. Perhaps the universal need will develop some intelligent professor who will teach us all how to make the best possible use of our hard-earned leisure; how to avoid the Scylla of exhausted legs and ruined stomachs on the one hand, and the Charybdis of finnicking and self-regarding pedantry on the other.

**Holiday Exercise.**

The Englishman's strong quality is energy, not discretion. He is better at doing than at thinking. More especially does he fail in thinking of what he regards as small and unimportant matters. Even when he is ill he has little respect for the physiologist and the doctor; when he is well he has no respect at all. That is not the doctor's fault, but the Englishman's. When the doctor also happens to be an Englishman, this indifference on the part of his fellow-countrymen rouses in him a sort of half-amused pugnacity. He is not, however, without a certain degree of sympathy with it. Still, he feels that he must pound away with his physiological cannon at the sandbags and other dull intellectual outworks of his unscientific neighbours in the hope that here and there a shot may enter and at least let in a little light. On the question of holiday exercise much has been said, but little has been laid to heart. The average Briton still thinks that he can leave his town office on the evening of one day, and after travelling one, or two, or three hundred miles to the place of his brief holiday sojourn, can get up next morning and walk twenty miles with advantage. He is even stupid enough to imagine that if he does not walk twenty the first day, and an additional mile or two on each succeeding day, he is not getting all the good he should out of his holiday. What a pity it is he does not know that he is exercising the brains of a sheep with the legs of a man! In London and other large towns we do not ordinarily walk more than three or four miles a day at the most. To jump from three or four to five times that distance is as discreet as it would be to spend five times one's income merely because we had removed to a new house, although the actual profits of our business remained practically the same. So far as exercise is concerned, the first day of a holiday should be a day of comparative dawdling. The breakfast should be late, not early. Between breakfast and lunch an hour may be spent in very gentle outdoor walking. After lunch a couple of hours should be taken on the sofa or an easy chair, until the time for afternoon tea arrives. Between afternoon tea and dinner another hour of gentle walking may be indulged in. After dinner a game at whist, or a concert if one be available, and then to bed. This for the first day. On the second a little more and a little sharper walking. The dawdling feeling must receive notice to quit. On the third day real exercise may begin; though even then no long excursion should be undertaken. If the holiday is to last for a month or longer, the first fortnight should be devoted to this gentle training. If, as at Easter, it is not to last for more than a few days or a week, the whole of the time should be spent in comparatively easy perambulations, and strenuous exercise should be avoided. Are we English then becoming an effeminate race? Not at all; but neither man nor horse can have either wind or legs whose life is spent in an office or a stable. Give us town men a little time to throw off our town legs and lungs, and we will walk, or drill, or hunt with the best. But we shall prove ourselves lunatics, not men of sense, if we rush from the office and the study and try to compete at once with our country cousins in muscle or in the power of using up oxygen. Every man to his trade. If the country cousin can beat us with his legs, we can beat him with our brains; at any rate until he has had time to get his into training.

